# Comprehensive strength evaluation system of commercial centres based on multi-source data: a case of Hefei central city

**DOI:** 10.1038/s41598-023-44139-x

**Published:** 2023-10-10

**Authors:** Jingyuan Chen, Zhiqiang Gan, Dan Li, Yunbin Zhang, Cheng Wang, Xiao Tao, Meng Zhu

**Affiliations:** School of Forestry and Landscape Architecture, Anhui Agricultural University, Hefei, 230000 China

**Keywords:** Psychology and behaviour, Environmental economics, Statistics

## Abstract

Urban commercial centres are the most concentrated areas of economic activity. Understanding the spatial distribution pattern and comprehensive strength of urban commercial centres is important to guide the reasonable graded allocation of urban commercial space, spatial structure optimization and sustainable development of the commercial economy. Herein, mobile phone signalling data are used to identify the functional connection between recreational and residential places and local spatial autocorrelation analysis is used to identify the 24 commercial centres in the central city of Hefei. The comprehensive strength evaluation system of commercial centres is constructed from their basic conditions and customer consumption behaviours, and their comprehensive strength indices are accordingly measured and graded. The spatial distribution characteristics of commercial centres at all levels are analysed, and optimisation suggestions are made for the whole area and region. The following conclusions are drawn from the results of this study. (1) Compared to traditional single-perspective evaluations, the developed comprehensive strength evaluation system considers supply and demand perspectives for commercial centres, providing a more holistic and accurate portrayal of the strengths of various centres within a region. (2) The current commercial centres are characterised by ‘large dispersion and small concentration’ in spatial and hierarchical distribution. (3) The commercial centres in Hefei have formed a relatively complete ‘first, second, and third level’ commercial centre system, with the first level as the core and relying on the urban road system to form a network spatial connection. (4) Most of the commercial centres are concentrated in the First Ring Road, Swan Lake in the Government Affairs District and Binhu Century Town Estate, while most areas north of the Second Ring Road and Binhu New District still lack large-scale commercial centres. This study provides a technical reference for analysing urban commercial spatial structure patterns and provides decision support for optimising the spatial layout of urban commercial service functions.

## Introduction

An urban commercial centre is an important carrier of urban vitality and an integral part of urban spatial structure. While promoting stock planning and construction, building a multi-level urban commercial centre system is vital in urban construction activities. Guiding urban polycentricity has become an important strategy and it is crucial for relieving the pressure on urban population, traffic and environment^1–3^. The study of commercial centre identification and its comprehensive strength at the city level is conducive to understanding the vitality level of a city, judging the rationality of urban spatial layout, and providing a basis for preparing urban territorial and spatial planning, residential area planning, commercial centre system planning, and traffic special planning.

Traditional commercial centre studies generally use the central place doctrine at the city level based on experience^4–6^ or random sampling^7^ to determine the target population and subsequently study its size^6^, service area^4,6^ composition of businesses^7^ and classification^7^. The applicability of traditional research methods diminishes with increasing size of the city. Furthermore, some scholars have attempted to analyse the shopping centre locations at the micro-community level from the perspective of the quality of retail supply to consumers and the effect of shopping on the environment^8^. However, research at the city level is challenging due to the limitations of traditional data access difficulties.

With the application of big data in urban and rural planning, some scholars have begun using big data such as POI (Point of Interest)^9^ and night-time light data^10^ to identify urban spatial structure^11^ and explore the connection among urban centres^12^, moreover, some scholars have used big data to further refine the functions of urban centres and study the spatial distribution of employment and commercial centres from the city level^13,14^. POI data have been used to analyse the spatial structure and clustering characteristics of urban commercial centres^15,16^, and further analyse their spatial and temporal evolution characteristics^17^. The spatial and temporal evolution characteristics of urban commercial centres in Shanghai and Dongguan were simulated by using taxi GPS data and localised contour tree method to delineate the boundaries of urban commercial centres^18,19^. A commercial spatial distribution map was generated using software location check-in data, and the density of commercial distribution is determined by using kernel density analysis^20^. These studies were mostly performed from the perspective of the basic conditions of the commercial centres, in which the strength of the commercial centres was quantitatively analysed according to the area of the commercial centres^21^, accessibility of traffic^22^ and number of POI^23,24^. Furthermore, with the enrichment of research data, some scholars have investigated the attractiveness of commercial centres to customers, i.e. customer consumption behaviour^25–29^. In this perspective, the strength of commercial centres is quantitatively analysed based on the number of customers per unit area^30^, total number of visits^31^ and actual service range^32^. However, the strength of a commercial centre is not only reflected in its own basic conditions or customers’ consumption behaviour, but also influenced by the combination of the two aspects. Most studies have been performed based on a single aspect, such as the basic conditions of the commercial centre or the customer’s consumption behaviour, without a comprehensive consideration of both the aspects.

Based on multi-source data, this study first identifies commercial centres in Hefei through data processing and spatial autocorrelation analysis, and subsequently establishes a new index system by combining the basic conditions of commercial centres and customer consumption behaviour. The comprehensive strength of each commercial centre is evaluated from four dimensions transportation conditions, service scale, visits and sphere of influence. The results are graded, and the distribution characteristics of commercial centres at different levels are described. Finally, targeted planning suggestions are proposed.

## Study area and data sources

### Scope of the study

**Figure 1:**
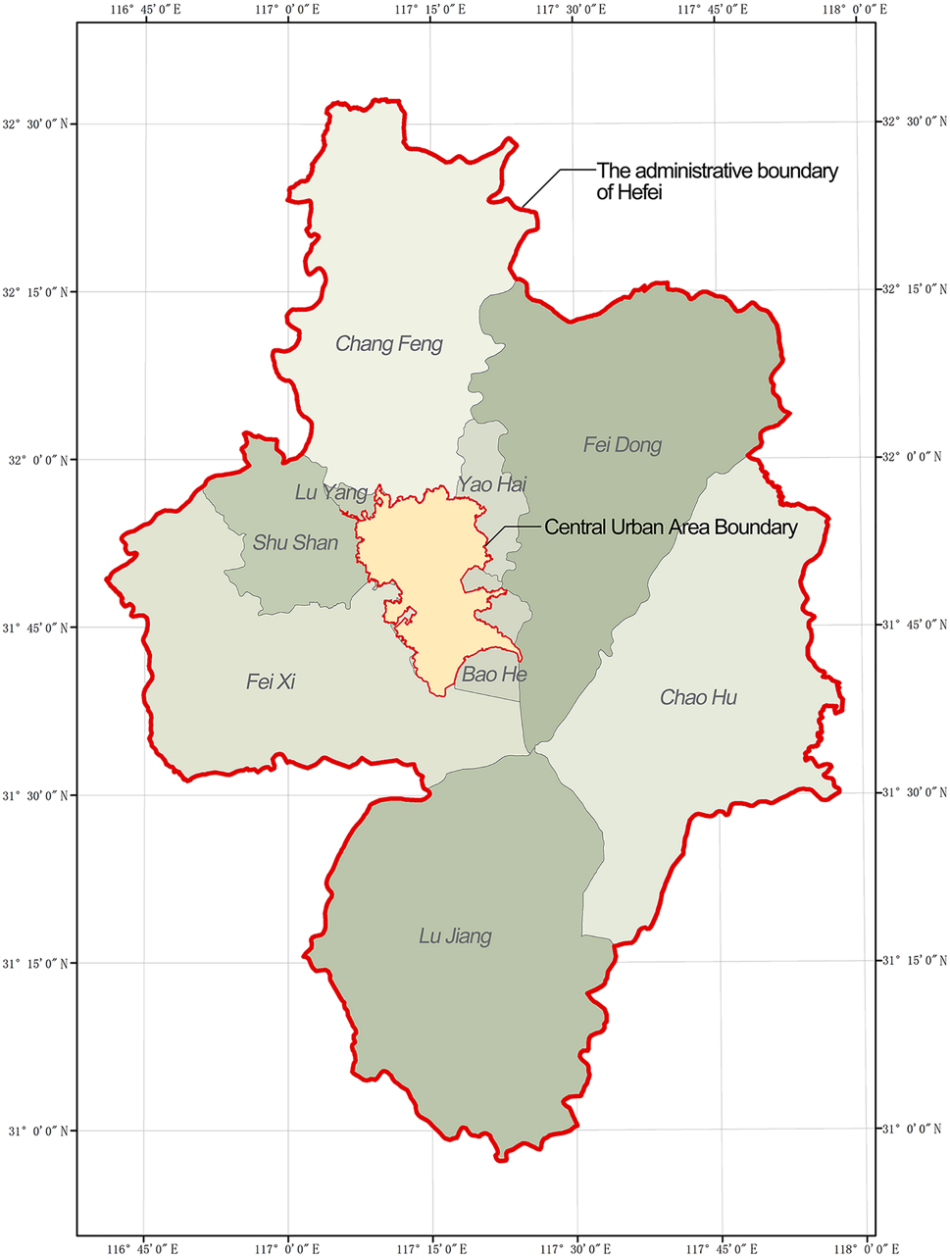
Scope of Hefei central city.

As a new first-tier city in the Yangtze River Delta city cluster, Hefei has experienced rapid commercial development and formed several large-scale commercial centres in the central urban area. This study selects the central urban area of Hefei as the research object. The research area is selected from the central urban area of the city delineated in ‘The Hefei Urban Master Plan (2011–2020)’, covering an area of 486.61 km^2^ (Fig. 1).

### Data sources and processing

#### Data sources

The study data include mobile phone signalling data, POI data, and road data in Hefei (Table 1). Among them, the mobile phone signalling data are obtained from the Unicom Smart Footprint data platform. The data used are the mobile phone signalling data (desensitised) of all Unicom users within the city limits of Hefei, Anhui Province, over 16 consecutive days (10 working days and 6 rest days) in May 2021. They are used to identify residential and recreational areas in the central urban area of Hefei, and subsequently filter the commercial centres and identify the number of people staying and the length of stay in each commercial centre. The road data are obtained from the Open Street Map of primary roads and secondary roads. The POI data are obtained from the Gaode open platform (https://lbs.amap.com/). The POI data of Hefei are crawled according to the classification of Gaode POI data, including food and beverage, shopping and consumption, leisure and entertainment, and bus and subway stations. The grid data required for studying commercial centre identification and the division of the sphere of influence performed in this study are obtained from a 250 m × 250 m fishing grid delineated based on the size of the base station grid, with 8,405 grids. The central urban area boundary is derived from “The Hefei Urban Master Plan (2011–2020)”.

**Table 1:** Data acquisition list.

Data name	Main content	Year	Data source
Administrative boundary data	Hefei central city boundary	2011	Hefei urban master plan (2011–2020)
Land use data	Present land use map	2020	Hefei urban master plan (2011–2020)
Satellite image data	Google satellite images	2021	Google earth
Mobile phone signalling data	Mobile phone signalling desensitisation data of all China Unicom subscribers in Hefei in May	2021	Unicom smart footprint
POI data	Gaode map data of various interest points	2021	Gaode open platform (https://lbs.amap.com/)
Road data	Road data of Hefei at all levels	2021	Open street map

#### Data processing

The mobile phone signalling data are processed to extract the user usage records within the central city of Hefei. Users who appear more than eight times in 16 days are marked as active users, and 2.81 million active users are identified, accounting for 29.7% of the total population of Hefei. By analysing the stay data of 16 days, the place where users stay for the longest duration and appear most frequently from 9:00 p.m. to 8:00 a.m. on the second day is marked as the residence^33^. Furthermore, the stay data of six rest days is analysed. Assuming that the opening hours of commercial centres are from 9:00 a.m. to 9:00 p.m., the location where users stay for more than half an hour during that period in six days was marked as a recreation place. A total of 930,000 users with identifiable residences are obtained, and 1.86 million recreation trips with identifiable recreation places are extracted, forming a table of recreation-residence function links (Table 2). The above rules are deductive in nature, and although there are certain errors, they can still reflect the commercial activity behaviour of residents and better reflect the travel relation between residences and commercial centres.

**Table 2:** Table of functional links between recreation and residence for a particular user.

User number	Date	Longitude and latitude of base station in the recreation area	Longitude and latitude of the base station at the place of residence	Start time of stay	End time of stay	Stay time (min)
1	05.15	31.819xxx,117.229xxx	31.735xxx,117.340xxx	10:38:44	11:53:04	75
1	05.15	31.837xxx,117.280xxx	31.735xxx,117.340xxx	14:23:24	16:01:35	38
1	05.15	31.855xxx,117.251xxx	31.735xxx,117.340xxx	19:59:02	21:45:12	106
1	05.16	31.864xxx,117.282xxx	31.735xxx,117.340xxx	13:15:42	14:08:50	53
1	05.16	31.846xxx,117.216xxx	31.735xxx,117.340xxx	18:57:41	20:54:44	117

## Research methodology

### Study design

The research design of this study is divided into four steps: first, the mobile phone signalling data extracted from the smart footprint are processed for identifying the residential and recreational places, and the residential and recreational trips are visualised using kernel density analysis to obtain the spatial distribution of residential and recreational trips. Second, the recreational trips of each base station are apportioned into the delimited grid. ‘Getis-Ord ${G}_{i}^{*}$ hot and cold analysis’ is applied to identify the hotspot areas for recreational activities. The identified hotspot areas are compared with the present land use and satellite image maps of Hefei to screen out commercial centres. Third, based on the existing research, a comprehensive strength evaluation system of commercial centres is established by combining the characteristics of commercial centres. Fourth, using the identified commercial centres, the data of each index of each commercial centre are separately counted, weights of each index are determined by the entropy weight method, and the comprehensive strength index of each commercial centre is calculated using the TOPSIS model. Fourth, the natural interruption point method is used to classify the commercial centres into three levels based on the size of the comprehensive strength index, and their spatial characteristics are analysed.

### Getis-Ord ${{\varvec{G}}}_{{\varvec{i}}}^{\boldsymbol{*}}$ cold and heat analysis

‘Getis-Ord ${G}_{i}^{*}$ hot and cold analysis’ proposed by Geddes^34^ and Ord^35^, is commonly used to describe the characteristics of local spatial cluster analysis. It scores the degree of aggregation of the specified variable on the local spatial region to get the z-score and p-value, and forms cold and hot spots according to the high and low z-score to distinguish the degree of aggregation of variables in space. If the z-score is positive, the higher the z-score, the tighter the hot spots formed. On the contrary, if the z-score is negative, the lower the z-score, the tighter the cold spots formed. The formula is presented as follows:

1 $${G}_{i}^{*}=\frac{{\sum }_{b=1}^{n}{w}_{ab}{x}_{b}-\overline{X}{\sum  }_{b=1}^{n}{w}_{ab}}{S\sqrt{\frac{\left[n{\sum }_{b=1}^{n}{w}_{ab}^{2}-{\left({\sum }_{b=1}^{n}{w}_{ab}\right)}^{2}\right]}{n-1}}},$$

where,

2 $$\overline{X }=\frac{{\sum }_{b=1}^{n}{x}_{b}}{n},$$

3 $$S=\sqrt{\frac{{\sum }_{b=1}^{n}{x}_{b}^{2}}{n}-{\left(\overline{X }\right)}^{2}},$$

The experiment divides the space of the research object into *n* basic spatial units, where ${G}_{i}^{*}$ calculates the z-score, ${x}_{b}$ represents the attribute value of unit* b*, and ${w}_{ab }$ is the spatial weight between unit *a* and *b*, and *n* is number of elements.

### Improved Huff’s gravity model

The gravity model proposed by Huff is suitable for calculating the probability between the origin and destination of residents’ trips, and applying it to the sphere of influence of commercial centres. It can accurately describe the strength of attraction and range of influence of commercial centres on the surrounding residential areas^36^. Herein, based on the improved Huff gravity model^37,38^, the distance between the origin and the destination is accurately measured by combining the route planning tool of Gaode Map. The model equation is as follows:

4 $${P}_{ef}=\frac{{S}_{f}{T}_{ef}^{-\zeta }}{{\sum }_{f=1}^{n}{S}_{f}{T}_{ef}^{-\zeta }},$$

5 $${\sum }_{f=1}^{n}{P}_{ef}=1,$$

where ${P}_{ef}$ represents the probability that residence *e* within the sphere of influence of commercial centre *f*, ${S}_{f}$ represents the total number of customer recreation in commercial centre *f*; ${T}_{ef}$ represents the distance between residence *e* and commercial centre *f*; ζ represents the distance attenuation coefficient, which is set to 2.5 based on empirical research findings from previous studies^37^; and *n* represents the number of commercial centres.

Herein, the important variable ${T}_{ef}$ in the model is proposed for optimisation. The optimal path distance from residence *i* to commercial centre *j* is simulated using the path planning tool of Gaode Map to achieve a more accurate model.

### Comprehensive strength evaluation system of commercial centres

To determine the comprehensive strength of the commercial centre, this paper draws on the existing research results^30,31,38^. The evaluation indices are determined through expert consultation, including two aspects: the basic conditions of the commercial centre and customers’ consumption behaviour. The basic conditions have an important influence on the attractiveness of a commercial centre and are a carrier of its ability to attract and gather customers^39^. Customer consumption behaviour is the whole process of customer behaviour from inducing consumption demand, selecting consumption places, making consumption travel and consuming. It is an indispensable perspective for examining the structure of urban commercial space and a representation of the actual operating condition of commercial centres^40^.

The basic conditions are mainly evaluated at two levels: transportation conditions and service scale. First, the accessibility of commercial centres is determined by the quality of transportation conditions. Residents in Hefei mostly use personal cars, buses and the subway to travel. Therefore, ‘density of primary and secondary roads’ and ‘density of public transportation stations’ are selected to measure the transportation conditions of the commercial centre. The higher the value, the better the transportation condition and the greater the comprehensive strength of the commercial centre. The density of public transportation stations is calculated by setting the average walking speed of customers at 4.8 km/h and the walking time at 5 min. The most suitable walking distance is calculated to be 400 m. Therefore, the statistics of the density of public transportation stations (bus stations and subway stations) within 400 m of the boundary of the commercial centre are calculated. Second, the size of the service scale also reflects the comprehensive strength of the commercial centre. The service scale mainly includes the area of the commercial centre and the richness of the business. Therefore, the ‘area of the commercial centre’ and ‘the number of POI per unit area’ are selected to measure the service scale of the commercial centre. The higher the value, the greater the comprehensive strength of the commercial centre.

Regarding customer consumption behaviour, the more visitors, the longer the customer stays, the larger the area of influence becomes, the more the commercial centre can meet the needs of more customers and the greater its comprehensive strength. Therefore, ‘visits per unit area’, ‘average length of stay’ and ‘absolute sphere of influence’ are selected to measure the ‘visits’ and ‘sphere of influence’ of a commercial centre. Among them, the absolute sphere of influence is based on the sphere of influence, which refers to the spatial unit of a commercial centre that is more attractive to residents in the grid than other commercial centres. It can truly reflect the main service area of the commercial centre^41^. According to the rank-size law^42^, for a grid cell attracted by 24 commercial centres, if the proportion of the most attractive commercial centre is higher than 26.48% and the proportion of the second most attractive commercial centre is less than one-half of it, then the grid belongs to the absolute sphere of influence of the most attractive commercial centre. However, a grid that does not satisfy this condition indicates that although the residents in this grid mainly visit the most attractive commercial centre, a larger share of them still go to other commercial centres. The grid becomes a power contention area for multiple commercial centres.

In summary, the evaluation index system comprises two sub-goal layers, four primary indicators and seven secondary indicators. Among them, the basic conditions include four secondary indicators of density of primary and secondary roads, density of public transportation stations, area of commercial centres and number of POI per unit area. The customer consumption behaviour includes three secondary indicators: number of visits per unit area, average length of stay and area of absolute sphere of influence. All seven indicators are positive, as shown in Table 3.

**Table 3:** Comprehensive strength evaluation index system of commercial centre.

Sub-target layer	Tier 1 indicators	Secondary indicators	Calculation method	Pointing	Weights
Self-base conditions	Transportation conditions	C1: density of public transportation stations (pcs/km^2^)	Number of public transportation stations/area of commercial centre	+	0.0663
		C2: density of primary and secondary roads (km/km^2^)	Length of primary and secondary roads/area of commercial centre	+	0.0358
	Service scale	C3: area of commercial centre (km^2^)	Derived from the results of Getis-Ord ${G}_{i}^{*}$ hot and cold analysis integrated with satellite imagery identification	+	0.2173
		C4: number of POI per unit area (pcs/km^2^)	Number of POI/area of commercial centre	+	0.1020
Customer consumption behavior	Visits	C5: visits per unit area (person/km^2^)	Visits/commercial centre area	+	0.1159
		C6: average length of stay (min)	Total customer length of stay/visits	+	0.0730
	Sphere of influence	C7: absolute sphere of influence area (km^2^)	Calculated from Eqs. (4) and (5)	+	0.3897

### Comprehensive strength index model of commercial centres

#### Entropy method

Herein, the entropy method is used to determine indicator weight. The entropy method objectively determines the weights through information calculation, effectively avoiding the bias caused by subjective assignment and making the results more objective^43^. The steps are as follows:

1. Construction of the Initial Assessment Matrix. This study constructs the original matrix using *m* entities and *n* indicators.
  6 $$X={\left[{x}_{ij}\right]}_{m\times n},$$
2. Data standardisation. Because of the different dimensions and orders of magnitude of each indicator, the original data of each indicator are standardised using the polarisation method to eliminate the influence of the indicators on the results. The standardised results are shifted by 0.01 to be between [0.01–1.01] to eliminate the influence of the ‘0’ value on the results,. Because all indicators in this study are positive, the standardisation formula is as follows:
  7 $${x}_{ij}^{\prime}=\frac{{x}_{ij}-\mathrm{min}({x}_{ij})}{\mathrm{max}\left({x}_{ij}\right)-\mathrm{min}({x}_{ij})}+0.01,$$
  where ${x}_{ij}$ represents the original value of the *j*th indicator of the *i*th commercial centre and ${x}_{ij}^{\prime}$ represents the value of the *j*th indicator of the *i*th commercial centre after standardisation.
3. Calculation of the information entropy value ${e}_{j}$ and information utility value ${d}_{j}$ of the indicator:
  8 $${y}_{ij}={x}_{ij}^{\prime}/\sum \limits_{i=1}^{m}{x}_{ij}^{\prime},$$
  9 $${e}_{j}=-\frac{1}{\mathrm{ln}\left(n\right)}\sum_{i=1}^{m}\left({y}_{ij}\mathrm{ln}{y}_{ij}\right),$$
  10 $${d}_{j}=1-{e}_{j},$$
  where ${y}_{ij}$ represents the share of the *i*th business centre value under the *j*th indicator, ${e}_{j}$ represents the information entropy value of the *j*th indicator and ${d}_{j}$ represents its information utility value. The larger the entropy value of an indicator, the lower its information utility, the smaller its role in the comprehensive evaluation and the smaller its weight.
4. Determination of indicator weights:
  11 $${w}_{j}={d}_{j}/\sum \limits_{i=1}^{n}{d}_{j},$$

#### TOPSIS model

The TOPSIS method is a ranking evaluation method that approximates the ideal value, and it is a commonly used multi-objective decision analysis method. This method involves the quantitative study and analysis of multiple indicators of multiple objects, selecting the ideal value of each indicator and then calculating the closeness of each indicator to the idealised target. It evaluates the relative merits of existing objects, and ranks the merits of multiple objects^44^. The TOPSIS method can objectively evaluate each object based on the value of the indicator, and reflects the gap between each object and the idealised target. The data used for the TOPSIS method herein are the standardised data, and the calculation steps are as follows:

1. Establish the weighted normal matrix R:
  12 $$R={\left\{{w}_{j}{P}_{ij}\right\}}_{m\times n},$$
  where ${w}_{j}$ represents the weight value of the *j*th indicator, ${P}_{ij}$ represents the data corresponding to the *j*th indicator of evaluation entity *i*, *n* represents the number of evaluation indicators (columns) and *m* represents the number of evaluation entities (rows).
2. Determine the ideal solution ${X}^{+}$ with the negative ideal solution ${X}^{-}$.
  13 $${X}^{+}=\left\{{x}_{1}^{+},{x}_{2}^{+},\ldots ,{x}_{j}^{+}\right\},$$
  14 $${X}^{-}=\left\{{x}_{1}^{-},{x}_{2}^{-},\ldots ,{x}_{j}^{-}\right\},$$
  where ${x}_{j}^{+}$ represents the optimal solution data for the *j*th indicator and ${x}_{j}^{-}$ represents the worst-case solution data for the *j*th indicator.
3. Calculate the distance of each indicator value from the positive and negative ideal solutions:
  15 $${D}_{i}^{+}=\sqrt{\sum_{j=1}^{n}{({x}_{j}^{+}-{x}_{ij})}^{2}},$$
  16 $${D}_{i}^{-}=\sqrt{\sum_{j=1}^{n}{({x}_{j}^{-}-{x}_{ij})}^{2}},$$
4. Calculate the closeness of each commercial centre to the ideal solution, i.e., the composite strength index ${C}_{i}$ of commercial centres.
  17 $${C}_{i}=\frac{{D}^{-}}{{D}^{+}+{D}^{-}}, 0<{C}_{i}<1,$$
  where the higher the ${C}_{i}$ value, the stronger the comprehensive strength of the commercial centre.

## Research results and analysis

### Commercial centres identification

**Figure 2:**
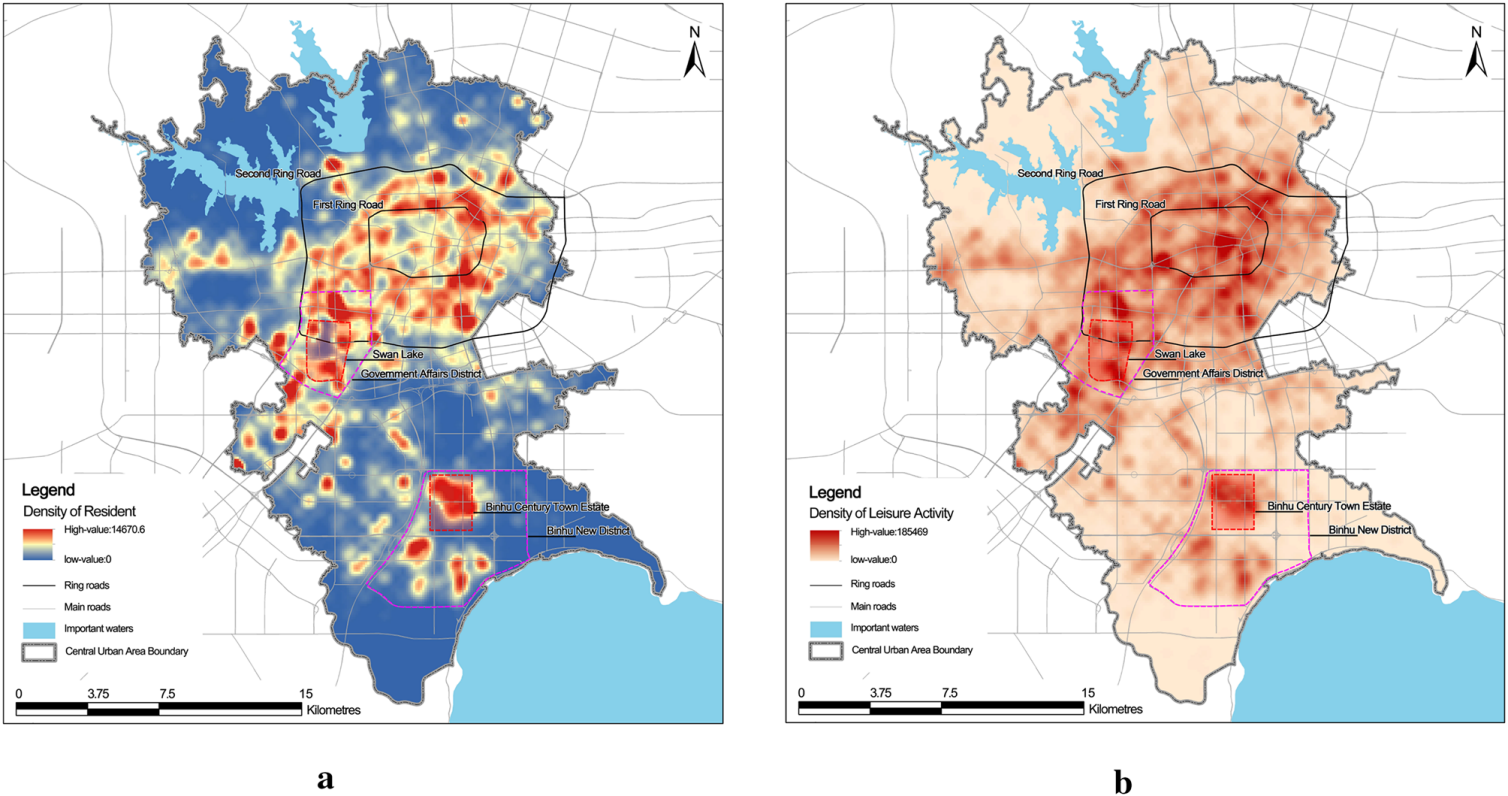
Residential density map of the central urban area (**a**) and leisure activity density map of the central urban area (**b**).

The identified 16 days of residence and 6 rest days of recreation trips are analysed using kernel density with each base station as coordinates. The search radius is set to 600 m to visualise the residence and recreation behaviour. The distribution of residence density and recreational activity intensity in the central city is obtained (Fig. 2). The recreational activities are primarily concentrated within the Second Ring Road, and the intensity of recreational activities decreases from the centre to the outside (Fig. 2b). However, because of the construction of Government Affairs District and Binhu New District, some gathering areas are found in the periphery of the centre, but the recreational activity intensity is weaker than in the central area.

**Figure 3:**
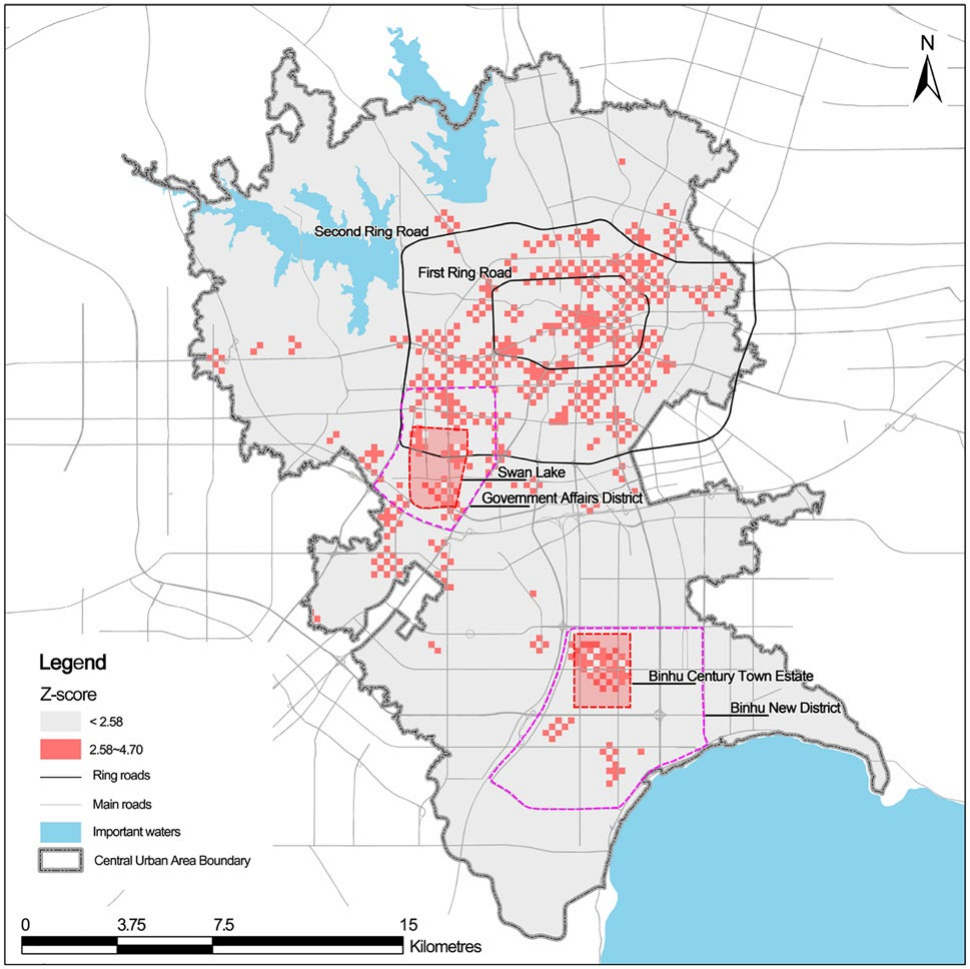
Hotspots for recreation activities.

**Figure 4:**
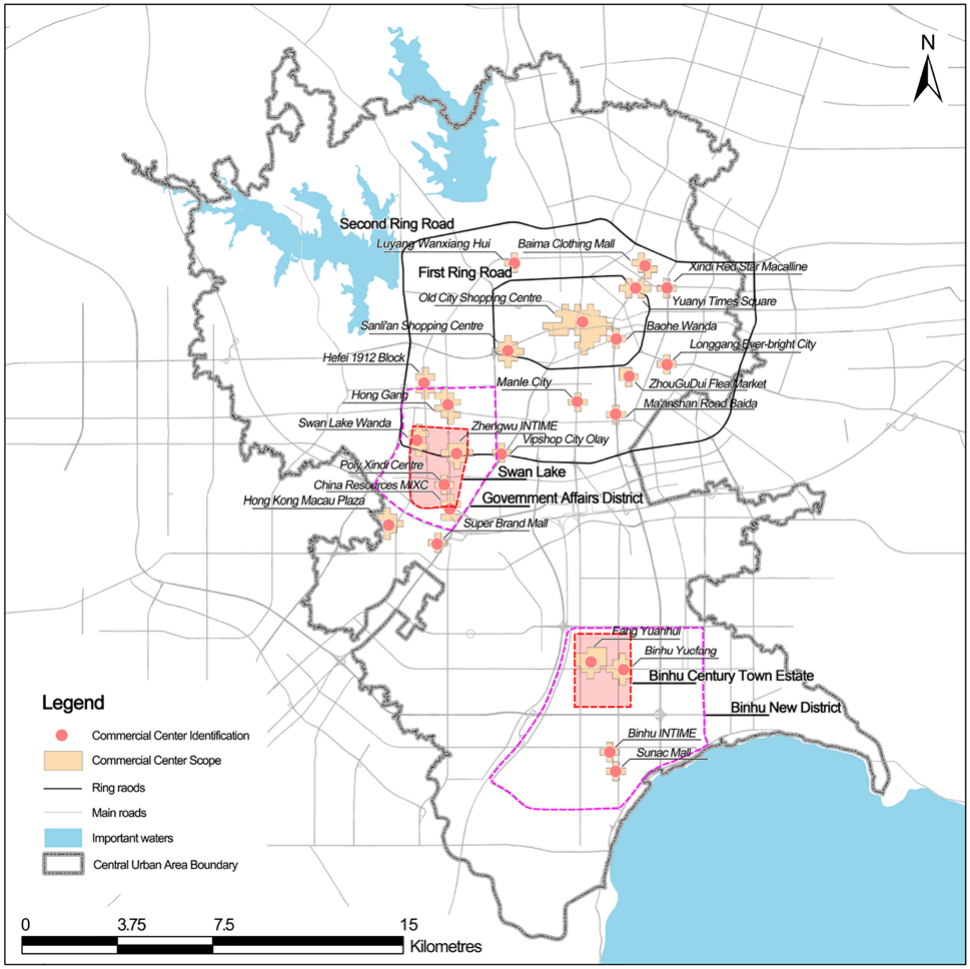
Commercial centres identification results.

Commercial centres are often highly concentrated areas of various recreational activities in the city, with considerable recreational trips. Therefore, to precisely identify the boundaries of commercial centres, the recreational trips of each base station are apportioned into a delimited 250 m × 250 m grid. Subsequently, through ‘the Getis-Ord ${G}_{i}^{*}$ hot and cold analysis’, the hotspot areas (Fig. 3) are selected at a 1% significance level (z-score > 2.58), indicating that the recreational activities of the residents in the six rest days exhibit significant high-value clustering characteristics in these areas. Subsequently, the identification results are compared with the present land use map of Hefei and the satellite image map^45^. The grid mainly containing activities was screened out by removing the gathering areas of recreational activities such as hospitals, transportation hubs and parks, and the grids primarily dominated by commercial activities were selected. These grids were utilised to delineate the boundaries of commercial centres and define the area within the identified commercial centre boundaries as entirely dedicated to commercial activities. Finally, 24 commercial centres were identified, including Sunac Mall, Binhu INTIME and Binhu Yuefang (Fig. 4). Although these commercial centres occupy only 2.90% of the land in the central city, they contain 48.31% of the recreation trips (Table 4).

**Table 4:** Identification results of commercial centres.

Serial number	Business centre name	Area (km^2^)	Recreation volume (million people)	Share of total recreation (%)	Serial number	Business centre name	Area (ha)	Recreation volume (million people)	Share of total recreation (%)
1	Sunac Mall	0.3125	2.87	1.55	13	Ma’anshan Road Baida	0.3125	38.97	2.09
2	Binhu INTIME	0.3125	0.82	0.44	14	Manle City	0.3125	0.77	0.41
3	Binhu Yuefang	0.7500	4.31	2.32	15	Hefei 1912 Block	0.5625	0.88	0.47
4	Fang Yuanhui	1.0625	6.61	3.55	16	ZhouGuDui Flea Market	0.3750	0.45	0.24
5	Super Brand Mall	0.3125	0.37	0.20	17	Longgang Ever-bright City	0.3125	2.36	1.27
6	Hong Kong Macau Plaza	0.6250	3.31	1.78	18	Sanli’an Shopping Centre	0.8125	8.39	4.51
7	China Resources MIXC	0.5000	3.84	2.06	19	Old City Shopping Centre	2.6875	18.59	9.99
8	Poly Xindi Centre	0.3125	0.39	0.21	20	Baohe Wanda	0.3125	3.31	1.78
9	Zhengwu INTIME	0.7500	5.05	2.72	21	Yuanyi Times Square	0.5625	3.30	1.77
10	Vipshop City Olay	0.3125	2.80	1.51	22	Xindi Red Star Macalline	0.3125	0.37	0.20
11	Swan Lake Wanda	0.6250	4.10	2.20	23	Baima Clothing Mall	0.5000	3.19	1.71
12	Hong Gang	0.8750	9.21	4.95	24	Luyang Wanxiang Hui	0.2500	0.69	0.37
						Total	14.0625	89.86	48.31

The area of each commercial centre results from the identification of commercial centres identified in the above study. Each commercial centre is formed by the radiation of multiple shopping centres.

In terms of spatial distribution, 50% of the commercial centres are located in and around the First Ring Road, occupying 48% of the total area. Meanwhile, along with the development and construction of the Government Affairs District and Binhu New District, commercial centres of a certain scale have been formed, primarily distributed around Swan Lake in the Government Affairs District and Binhu Century Town Estate in the Binhu New District. The commercial centre is unidentified in most areas north of the Second Ring Road and Binhu New District. Therefore, the spatial distribution of commercial centres is centripetal clustering, and the multi-centre characteristics are obvious.

In terms of the total amount of recreational activities, the commercial centres in and around the First Ring Road account for 50.4% of the total amount of recreational activities in each commercial centre, Government Affairs District accounts for 33.33% and Binhu New District accounts for 12.67% (Table 4). Therefore, although recreational activities within commercial centres in Hefei City are dispersed across the entire domain, they demonstrate a concentrated pattern within three specific regions: the First Ring Road, Government Affairs District, and Binhu New District. This concentration indicates a clear multi-centric characteristic of the commercial centres.

The multi-centre characteristics of commercial centres in Hefei are more obvious, which may be because Hefei vigorously promotes the construction of the Government Affairs District and Binhu New District, and evacuates some functions of the Old City. However, because of the late start of the construction of Binhu New District, although some large-scale commercial centres already exist, some areas still lack commercial centres. Meanwhile, the urban construction activities north of the Second Ring Road are slow, and there is no large-scale commercial centre at present.

### Evaluation of the comprehensive strength of commercial centres

#### Determination of the comprehensive strength indicators of commercial centres

**Figure 5:**
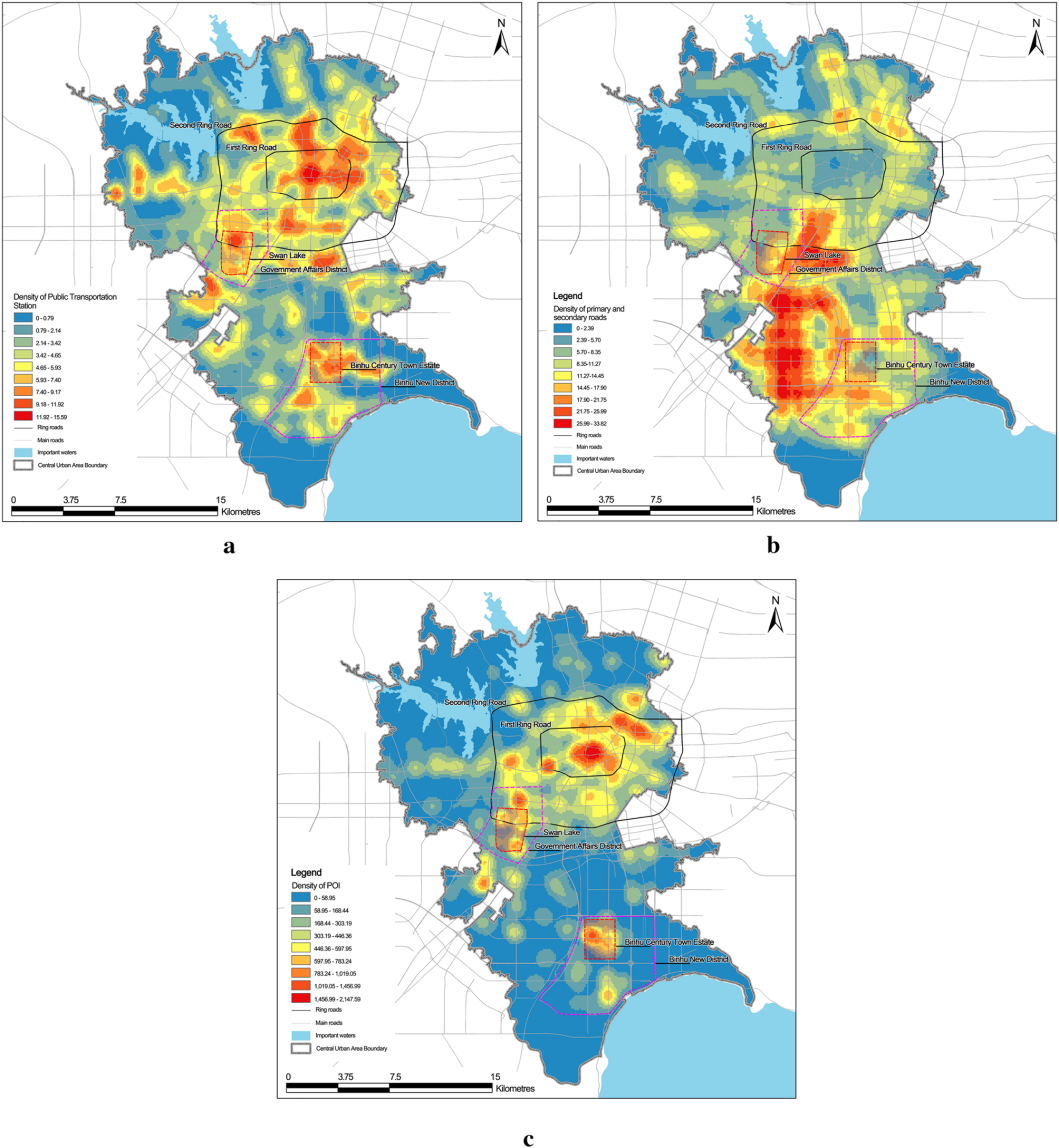
Density of public transport stations in the central urban area (**a**), primary and secondary roads in the central urban area (**b**), and POI in the central urban area (**c**).

Based on the seven selected indicators herein, the comprehensive strength parameters of the commercial centres are quantitatively calculated. Figure 5 depicts the nuclear density analysis of public transportation stations, number of major and minor arterial roads and number of POI in the central urban area of Hefei. These parameters are spatially overlaid with the above identified commercial centres to calculate the density of public transportation stations, density of primary and secondary roads and the number of POI per unit area for each commercial centre, corresponding to the transportation conditions and service scale in the evaluation system.

**Figure 6:**
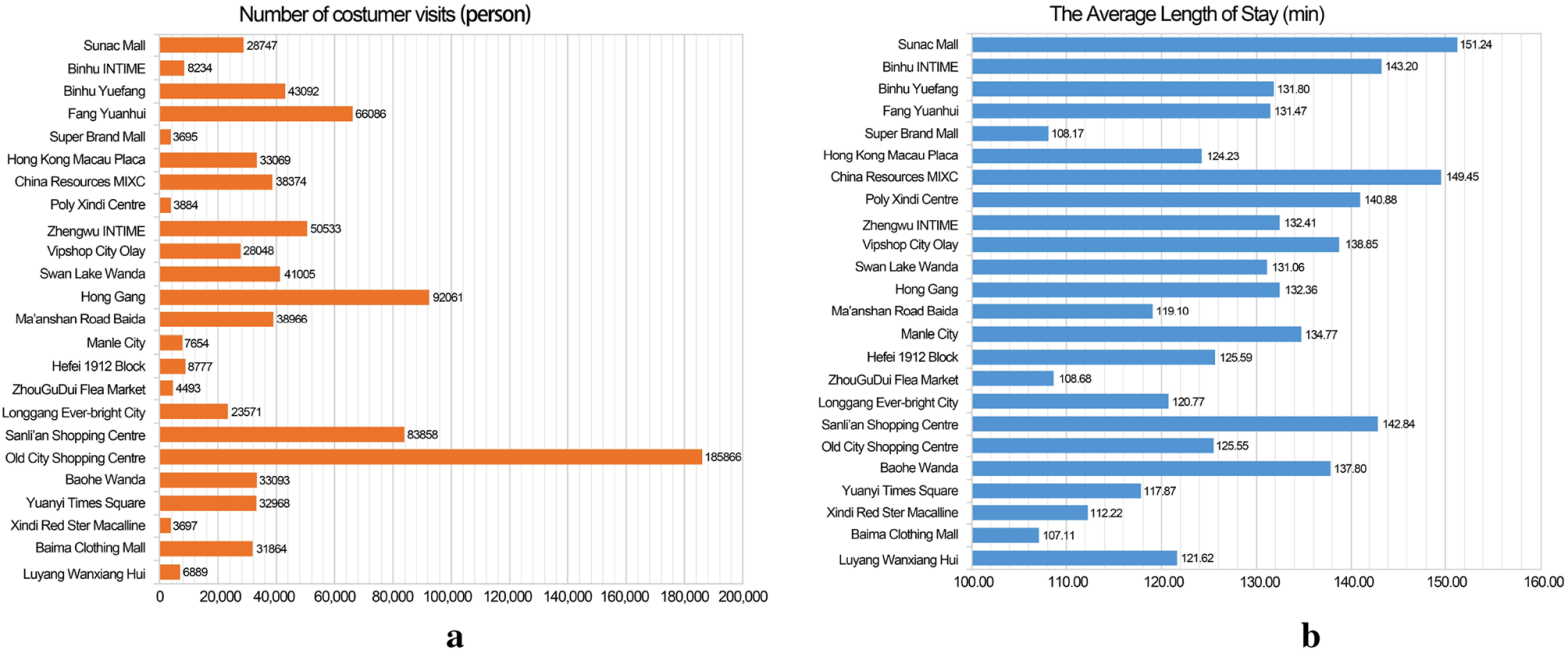
Number of customer visits in each commercial centre (**a**) and the average length of stay in each commercial centre (**b**).

The number of visits and the length of stay of customers are important indicators for the comprehensive evaluation of commercial centres. By separately counting the number of visits to each commercial centre (Fig. 6a), we observe that the Old City Shopping Centre, which is the centre of the Old City of Hefei, has large commercial places such as Huaihe Road Pedestrian Street and Hefei Yintai Centre, which are considerably attractive to customers; Meanwhile, commercial centres such as Sanli’an Shopping Centre, Hong Gang and Fang Yuanhui have more customer visits in a larger area and stronger attraction. Measuring the average dwell time of customers in each commercial centre (Fig. 6b), reveals that customers stay longer in Sunac Mall, Binhu INTIME, China Resources MIXC and Sanli’an Shopping Centre with an average stay of > 140 min, and shorter in Super Brand Mall, ZhouGuDui Flea Market and Baima Clothing Mall. The above data are processed and the number of visits per unit area and the average length of stay in each commercial centre are separately counted, corresponding to the indicators of visits in the evaluation system.

The absolute sphere of influence of each commercial centre is presented using the improved gravity model. Taking the grid 5079, the attractiveness of each commercial centre to the residents in this grid is calculated using the gravity model, and the results are presented in Table 5. The Sanli’an Shopping Centre exhibits has the highest attractiveness, accounting for50.25%. The second highest attractiveness is the Old City Shopping Centre accounting for17.53%, which is less than one-half of the Sanli’an Shopping Centre. Therefore, this grid is within the absolute sphere of influence of the Sanli’an Shopping Centre.

**Table 5:** Percentage of the attractiveness of each commercial centre to residents in grid 5079.

Name	Attractiveness ratio (%)	Name	Attractiveness ratio (%)	Name	Attractiveness ratio (%)	Name	Attractiveness ratio (%)	Name	Attractiveness ratio (%)
Sunac Mall	0.07	Hong Kong Macau Plaza	0.33	Swan Lake Wanda	1.06	ZhouGuDui Flea Market	0.17	Yuanyi Times Square	0.64
Binhu INTINME	0.02	China Resources MIXC	0.77	Hong Gang	16.19	Longgang Ever-bright City	0.66	Xindi Red Star Macalline	0.06
Binhu Yuefang	0.18	Poly Xindi Centre	0.11	Ma’anshan Road Baida	0.92	Sanli’an Shopping Centre	50.25	Baima Clothing Mall	0.53
Fang Yuanhui	0.34	Zhengwu INTIME	4.02	Manle City	0.54	Old City Shopping Centre	17.53	Luyang Wanxiang Hui	0.77
Super Brand Mall	0.05	Vipshop City Olay	2.10	Hefei 1912 Block	0.83	Baohe Wanda	1.84		

**Figure 7:**
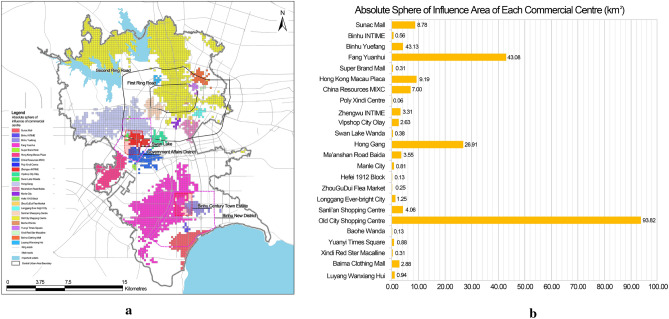
Absolute sphere of influence of commercial centres (**a**) and the absolute sphere of influence area of each commercial centre (**b**).

Thus, the central city is divided into the absolute sphere of influence of 24 commercial centres by removing the grid within the contested area of multiple commercial centres (Fig. 7). The absolute sphere of influence of commercial centres in the Old City occupies most of the area in the First Ring Road. The number of commercial centres in the Second Ring Road and the Government Affairs District is large and the distance between commercial centres is relatively small, causing fierce competition. The absolute sphere of influence of commercial centres is small and entirely in their surrounding areas. The absolute sphere of influence of some commercial centres is limited by the competition from other commercial centres; therefore, the absolute sphere of influence basically only remains within themselves and adjacent areas. Because of the late start of construction and small number of commercial centres in Binhu New District, the absolute sphere of influence of the commercial centres is generally larger than those of the commercial centres in other areas, reflecting the lack of large-scale commercial centres in Binhu New District.

#### Evaluation of comprehensive strength of commercial centres

The data of seven evaluation indicators of 24 commercial centres in Hefei are normalised according to Eq. (7). The processed data range from 0.01 to 1.01, of which 0.01 and 1.01 represent the lowest and highest levels, respectively. The normalised data are shown in Table 6.

**Table 6:** Normalised values of each indicator for each commercial centre.

Serial number	Name	Self-base conditions				Customer consumption behaviour		
		Transportation conditions		Service scale		Visits		Sphere of influence
		C1: density of public transportation stations	C2: density of primary and secondary roads	C3: area of commercial centre	C4: number of poi per unit area	C5: visits per unit area	C6: average length of stay	C7: absolute sphere of influence area
1	Sunac Mall	0.3964	0.5714	0.0346	0.6380	0.7203	1.0100	0.1030
2	Binhu INTIME	0.4840	0.4311	0.0346	0.0100	0.1387	0.8278	0.0153
3	Binhu Yuefang	0.5495	0.4188	0.2149	0.2946	0.4143	0.5695	0.0534
4	Fang Yuanhui	0.1201	0.4233	0.3420	0.5088	0.4563	0.5620	0.4688
5	Super Brand Mall	0.2210	0.6240	0.0346	0.0342	0.0100	0.0340	0.0127
6	Hong Kong Macau Plaza	0.4701	0.2240	0.1657	0.4037	0.3740	0.3979	0.1074
7	China Resources MIXC	0.3140	0.4802	0.1125	0.6267	0.5852	0.9694	0.0840
8	Poly Xindi Centre	0.3964	0.2925	0.0346	0.2354	0.0154	0.7752	0.0100
9	Zhengwu INTIME	0.2234	0.4258	0.2149	0.4335	0.5022	0.5833	0.0447
10	Vipshop City Olay	0.4840	0.9170	0.0346	0.3933	0.7005	0.7292	0.0374
11	Swan Lake Wanda	0.3407	0.5784	0.1657	0.4770	0.4865	0.5527	0.0134
12	Hong Gang	0.2250	0.5626	0.2682	0.5163	0.8374	0.5822	0.2964
13	Ma’anshan Road Baida	0.8347	0.7118	0.0346	0.3949	1.0100	0.2817	0.0472
14	Manle City	0.3964	0.5714	0.0346	0.4641	0.1222	0.6368	0.0180
15	Hefei 1912 Block	0.1897	0.6346	0.1370	0.2456	0.0435	0.4288	0.0107
16	ZhouGuDui Flea Market	0.2510	0.0100	0.0633	0.4281	0.0114	0.0456	0.0120
17	Longgang Ever-bright City	0.2210	0.3082	0.0346	0.1662	0.5735	0.3195	0.0227
18	Sanli’an Shopping Centre	0.0100	0.6328	0.2395	0.7011	0.8197	0.8197	0.0527
19	Old City Shopping Centre	0.2236	0.6504	1.0100	0.6899	0.5180	0.4279	1.0100
20	Baohe Wanda	1.0100	0.7205	0.0346	1.0100	0.8435	0.7054	0.0107
21	Yuanyi Times Square	0.6750	0.6153	0.1370	0.2179	0.4245	0.2538	0.0187
22	Xindi Red Star Macalline	0.8347	1.0100	0.0346	0.5382	0.0101	0.1258	0.0127
23	Baima Clothing Mall	0.5857	0.4451	0.1125	0.9267	0.4704	0.0100	0.0401
24	Luyang Wanxiang Hui	0.2596	0.3486	0.0100	0.2222	0.1494	0.3388	0.0194

Using Eq. (11), the weights of the indicators of the comprehensive strength of 24 commercial centres in Hefei are calculated (Table 3). The results reveal that the weights from high to low are C7 (0.3897), C3 (0.2173), C5 (0.1159), C4 (0.1020), C6 (0.0730), C1 (0.0663) and C2 (0.0340).

After the indicator weights are determined, the weighted normal matrix for evaluating the comprehensive strength of commercial centres in Hefei is constructed using Eq. (12). Subsequently, using Eqs. (15) and (16), the Euclidean distances from the comprehensive strength of the 24 commercial centres to the positive and negative ideal solutions are determined as follows: ${D}_{i}^{+}$= (0.447, 0.466, 0.411, 0.333, 0.467, 0.433, 0.442, 0.467, 0.427, 0.454, 0.438, 0.409, 0.444, 0.466, 0.458, 0.465, 0.458, 0.366, 0.091, 0.446, 0.439, 0.467, 0.437, 0.466); ${D}_{i}^{-}$ = (0.116, 0.071, 0.098, 0.155, 0.033, 0.078, 0.124, 0.064, 0.094, 0.106, 0.083, 0.127, 0.136, 0.060, 0.050, 0.048, 0.081, 0.157, 0.460, 0.133, 0.086, 0.079, 0.104, 0.043).

**Figure 8:**
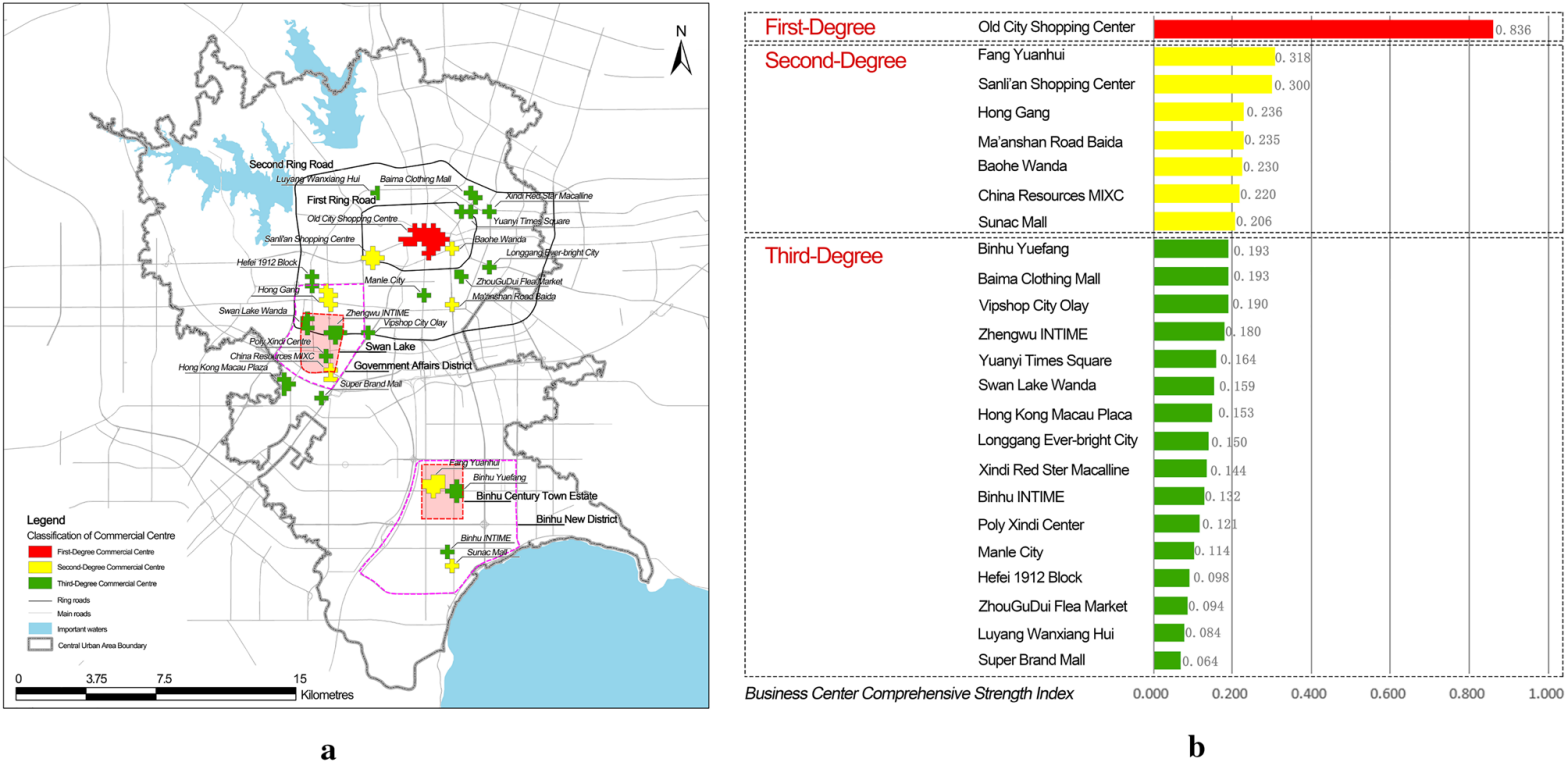
Spatial distribution of three levels of commercial centres (**a**), the comprehensive strength index and the classification of commercial centre (**b**).

The value of the comprehensive strength index ${C}_{j}$ of commercial centres is calculated using Eq. (17) (Table 7). Based on the ${C}_{j}^{+}$ value, each commercial centre is divided into three classes: [0, 0.2), [0.2, 0.5), and [0.5, 1], corresponding to the first, second, and third level commercial centres, respectively (Fig. 8).

**Table 7:** ${D}_{i}^{+}$, ${D}_{i}^{-}$ and ${C}_{j}$ values of each commercial centre.

Business centre	Positive ideal solution distance ${D}_{i}^{+}$	Negative ideal solution distance ${D}_{i}^{-}$	Comprehensive strength index ${C}_{j}$	Sort results
Sunac Mall	0.447	0.116	0.206	8
Binhu INTIME	0.466	0.071	0.132	18
Binhu Yuefang	0.411	0.098	0.193	9
Fang Yuanhui	0.333	0.155	0.318	2
Super Brand Mall	0.467	0.033	0.067	24
Hong Kong Macau Plaza	0.433	0.078	0.153	15
China Resources MIXC	0.442	0.124	0.220	7
Poly Xindi Centre	0.467	0.064	0.121	19
Zhengwu INTIME	0.427	0.094	0.18	12
Vipshop City Olay	0.454	0.106	0.19	11
Swan Lake Wanda	0.438	0.083	0.159	14
Hong Gang	0.409	0.127	0.236	4
Ma’anshan Road Baida	0.444	0.136	0.235	5
Manle City	0.466	0.06	0.114	20
Hefei 1912 Block	0.458	0.05	0.098	21
ZhouGuDui Flea Market	0.465	0.048	0.094	22
Longgang Ever-bright City	0.458	0.081	0.15	16
Sanli’an Shopping Centre	0.366	0.157	0.3	3
Old City Shopping Centre	0.091	0.46	0.836	1
Baohe Wanda	0.446	0.133	0.23	6
Yuanyi Times Square	0.439	0.086	0.164	13
Xindi Red Star Macalline	0.467	0.079	0.144	17
Baima Clothing Mall	0.437	0.104	0.193	10
Luyang Wanxiang Hui	0.466	0.043	0.084	23

Overall, the commercial centres in Hefei present a relatively complete ‘first-degree, second-degree and third-degree’ commercial centre system, with the first-degree commercial centre as the core, in cooperation with the surrounding second-degree and third-Degree commercial centres, forming a network-like spatial connection by relying on the urban road system, forming a ‘large scattered, small concentrated’ spatial layout feature. Each level of commercial centres is distributed in most areas of the central city, but relatively concentrated in the First Ring Road, Swan Lake in the Government Affairs District and the surroundings of the Binhu Century Town Estate. This feature is particularly obvious in high-grade commercial centre.

1. The core location of the first-degree commercial centre is outstanding, and the commercial facilities are relatively complete. The historical status of the Old City Commercial Centre has laid a good foundation for its development, while its superior transportation conditions, immense service scale and rich business structure attract many customers, forming a large sphere of influence and attraction, prompting its influence and attraction to be much higher than the surrounding areas, forming the only first-degree commercial centre.
2. There are seven second-degree commercial centres, which are formed gradually along with the urban expansion of Hefei and have a certain attraction in some surrounding areas. However, they are influenced by the first-degree commercial centres and the competition from several commercial centres with similar comprehensive strengths, resulting in a certain limitation of their scale. Taking Sanli’an and Baohe Wanda Commercial Centre as an example, although their commercial facilities are relatively complete, their comprehensive strength is still limited because of their proximity to the Old City Shopping Centre. Furthermore, Zhengwu INTIME, Swan Lake Wanda, China Resources MIXC and Vipshop City Olay are located near Swan Lake. Their commercial volume and transportation conditions are similar, which makes them attractive as a whole. However, because of their mutual competition and mutual limitations, individuals have similar comprehensive strengths. Only the comprehensive strength of a China Resources MIXC reaches the standard of second-degree commercial centre.
3. There are 16 third-degree commercial centres, which mainly serve the residents in the surrounding area with a small service scope. The aggregation effect is not large enough because of the influence of location, surrounding construction, surrounding living conditions and their facility conditions, such as Luyang Wanxiang Hui, Longgang Ever-bright City, and Manle City. In addition, some tertiary commercial centres are formed mainly by the surrounding special environment. For example, Baima Clothing Mall relies on the superior transportation conditions of Hefei Railway Station and Bus Station. Hong Kong Macao Plaza relies on the nearby university town. Meanwhile, some tertiary commercial centres provide commercial services for some residents because of their single business structure, such as Xindi Red Star Macalline, which mainly sells furniture, and ZhouGuDui Flea Market, which is mainly wholesale products.

## Discussion and conclusion

### Further discussion

With the advancement of urban stock planning, mastering the existing spatial structure system of commercial centres in cities is particularly important. Most traditional research methods for the spatial structure of commercial centres are based on the hierarchy of commercial facilities determined by existing plans^6,7^ or according to their conditions^46^. Amidst the current improvements in commercial centre service quality and transportation infrastructure, the emphasis on commercial centre size in these methods no longer remains the sole criterion for their categorisation nor fully represents the attractiveness of a commercial centre to customers. The continued use of these methods can lead to the oversight of factors such as store quality, service levels and supporting facilities, resulting in substantial evaluation errors. The accurate measurement of a commercial centre's appeal to customers has become a prominent topic among subsequent scholars. For example, Dolega et al. directly developed a production-constrained model from the customer perspective, attempting to simulate shopping mall foot traffic to categorise commercial centres based on their customer appeal^47^. However, simulating foot traffic inherently contains some degree of error. With the application of big data in urban planning and related domains, precisely understanding consumer travel patterns becomes possible. Mao et al. built on Dolega’s work, utilising datasets from taxi traffic to construct an ‘Attractiveness Degree Model’ that estimates customers’ tendencies to visit various commercial centres, categorising them hierarchically^27^. Although these studies adopt an innovative customer-centric approach to measuring commercial centres' comprehensive strength, their focus solely on travel intentions does not entirely capture customer consumption behaviour. Customer dwell time can also indicate a centre's attractiveness. Utilising a wider range of big data for a more detailed depiction of customer consumption behaviour is necessary. Furthermore, these studies categorise commercial centres purely in terms of customer travel desires, overlooking the centres' intrinsic attributes. This oversight can lead to situations where smaller centres cannot fulfil the functions of higher-level ones, contradicting real-world conditions. Thus, unlike the single-aspect methods based on a centre's intrinsic attributes or customer consumption behaviour, this study combines both perspectives. It utilises sources such as mobile signalling data and POI to evaluate and categorise commercial centres based on a synthesis of consumer demand and centre supply. This approach accurately captures consumer consumption intentions and reflects centre service capabilities, providing a comprehensive assessment of a centre's strength from supply and demand aspects. It offers a more accurate portrayal of consumer behaviour than traditional methods and considers the centres' developmental conditions. The spatial structure analysis of commercial centres in the central urban area of Hefei indicates that this study recognises more commercial centres and calculates their comprehensive strengths more reasonably than investigations solely relying on Baidu heatmap calculations^48^. This result demonstrates the higher accuracy of the proposed comprehensive strength evaluation system for commercial centres. This study not only provides a basis for grasping the hierarchy and spatial distribution characteristics of urban commercial centres, but also provides a basis for subsequent research on urban commercial centres.

Although this study covers both dimensions of commercial centres’ basic conditions and customers’ consumption behaviour, it lacks in-depth research on both dimensions. In terms of their basic conditions, POI data can be used for further analysis to classify commercial centres and study the relationship between their rank and type. Regarding customer consumption behaviour, this study only describes the displacement of citizens in space and time, and lacks the identification of citizens’ consumption behaviour in small areas within commercial centres, which is somewhat deductive. In the future, we can combine traditional data such as questionnaire surveys to refine the research on the social attributes of citizens and further study their preference for commercial centres from the perspective of consumers. We can also combine POI with questionnaire surveys to study the interaction between commercial centre types and consumer preferences. In the future, big data and traditional data can be combined to promote the rationalisation of the hierarchical allocation and spatial layout of commercial centres.

### Main conclusions

Commercial centres have always been vital for urban space, an important carrier of urban vitality, and the most concentrated area of economic activity. The mastery of the comprehensive strength of commercial centres and the classification of grades helps to understand the development status of commercial centres, grasp the level of urban commercial activities, and then provide a basis for adjusting the graded configuration of urban commercial space and guiding the rationalisation of urban spatial structure. First, this study identifies the residential and recreational areas in the central city of Hefei through mobile phone signalling data. Local spatial autocorrelation analysis is applied to identify commercial centres, and it is found that they show the multi-centre structural characteristics, but the aggregation trend is more obvious in the whole area. Second, the comprehensive strength evaluation index system of commercial centres is constructed from two levels of commercial centres’ basic conditions and customers’ consumption behaviour. The comprehensive strength index of commercial centres is measured by combining POI and road data, and the commercial centres are divided into three levels according to the size of the comprehensive strength index.

The study results show that the spatial and hierarchical distribution of the current commercial centres in the central city of Hefei is characterised by ‘large dispersion and small concentration’, and a more complete three-level commercial centre system has been formed. The multi centre characteristics are obvious. Most of them concentrated around the First Ring Road, Swan Lake in the Government Affairs District and Binhu Century Town Estate. This distribution is closely related to the development policy of Hefei, which recently has been vigorously promoting the construction of the Government Affairs District and Binhu New District to relieve the political, economic, residential and employment pressure in the Old City, gradually forming a new development nucleus. However, some areas still lack commercial centres in the whole area of Hefei commercial centre, such as north of the Second Ring Road and most areas in Binhu New District. At the same time, there are some clusters of commercial centres of the same level. Although it can improve the comprehensive commercial attractiveness of the area, the homogeneity of the industry will also cause competition and convergence among commercial centres, which is not conducive to the formation of higher-level commercial centres. Therefore, priority should be given to the layout of commercial facilities in areas lacking commercial centres to meet the needs of nearby residents, and scientific planning should be conducted to guide their business types and scales, to avoid the unreasonable phenomenon that the scale of commercial centres is too large or too small. Multiple secondary and tertiary commercial centres should be formed in the whole region. At the same time, the scale and business structure should be adjusted according to the supply and demand situation of each commercial centre and the area to form several first-grade or higher-grade commercial centres throughout the whole region, improve the grading configuration standards of commercial centres, and meet the diversified commercial facilities needs of the public. This study can provide a reference for the construction and planning of commercial centres in the city by mastering the characteristics of the commercial centre hierarchy and spatial distribution.

## Data Availability

The datasets used and/or analysed in the current study are not publicly available because of General Data Protection Regulations; however, they are available from the corresponding author upon reasonable request.

## References

[1] Huang D, Liu Z, Zhao X (2015). Monocentric or polycentric? The urban spatial structure of employment in Beijing. Sustainability.

[2] Huang DQ, Wan W, Dai TQ, Liang JS (2011). Assessment of industrial land use intensity: A case study of Beijing economic-technological development area. Chin. Geogr. Sci..

[3] Liu X, Derudder B, Wang M (2017). Polycentric urban development in China: A multi-scale analysis. Environ. Plan. B Urban Anal. City Sci..

[4] Dunn R, Wrigley N (1985). Beta-logistic models of urban shopping center choice. Geogr. Anal..

[5] Honda Y, Ohyama I, Kitamura S (1986). A study on a method of a index that indicates the activity of the shopping center in a city. Infrastructure Plan. Rev..

[6] Wang D (2015). Comparison of business districts in different levels of commercial centers in shanghai based on mobile phone signaling data: Taking Nanjing East Road, Wujiaochang, and Anshan Road as examples. Urban Plan. Forum.

[7] Ning YM, Huang SL (2005). The hierarchical system and its changing characteristics of commercial centers in Shanghai urban area. Areal Res. Dev..

[8] Yang ZZ (2002). Microanalysis of shopping center location in terms of retail supply quality and environmental impact. J. Urban Plan. Dev. ASCE.

[9] Zhou N (2022). Research on urban spatial structure based on the dual constraints of geographic environment and POI big data. J. King Saud. Univ. Sci..

[10] Cai JX, Huang B, Song YM (2017). Using multi-source geospatial big data to identify the structure of polycentric cities. Remote Sens. Environ..

[11] Zhou YQ, He X, Zhu YT (2022). Identification and evaluation of the polycentric urban structure: An empirical analysis based on multi-source big data fusion. Remote Sens..

[12] Hu XY, Yang HY, Yang JY, Zhang ZH (2019). Spatial correlation network of format in the central districts of a megacity: The case of Shanghai. Sustainability.

[13] Campos RBA, Chagas ALS (2021). Employment sub-centers of a megacity in a developing country: The case of the Municipality of São Paulo, Brazil. Nova Economia.

[14] Arribas-Bel D, Sanz-Gracia F (2014). The validity of the monocentric city model in a polycentric age: US metropolitan areas in 1990, 2000 and 2010. Urban Geogr..

[15] Han Z, Song W (2020). Identification and geographic distribution of accommodation and catering centers. Isprs Int. J. Geo-Inf..

[16] Yu L, Yu T, Wu YX, Wu GD (2020). Rethinking the identification of urban centers from the perspective of function distribution: A framework based on point-of-interest data. Sustainability.

[17] Chen SL, Tao HY, Li XL, Zhuo L (2018). Detecting urban commercial patterns using a latent semantic information model: A case study of spatial-temporal evolution in Guangzhou, China. PLoS ONE.

[18] Sun MQ, Fan HC (2021). Detecting and analyzing urban centers based on the localized contour tree method using taxi trajectory data: A case study of Shanghai. Isprs Int. J. Geo-Inf..

[19] Li HB, Xu XC, Li X, Ma SF, Zhang HH (2021). Characterizing the urban spatial structure using taxi trip big data and implications for urban planning. Front. Earth Sci..

[20] Hu QW, Wang M, Li QQ (2014). Urban hotspot detection and commercial area analysis based on check-in data using exploratory spatial data analysis. Acta Geodaetica et Cartographica Sinica.

[21] Wang F, Gao X, Xu Z (2015). Identification and classification of urban commercial districts at block scale. Geogr. Res..

[22] Han G, Feng XL, Kang RK, Jiang LL (2021). Identification and spatial structure characteristics of commercial centers in river network cities: A case study of Huai'an City Jiangsu Province. Areal Res. Dev..

[23] Meng-Jie Z, En-Jia Z, Zhuo-Ran S (2019). Research on the identification of multiple types of commercial center and spatial patterns in Wuhan based on POI data. South Architect..

[24] Zeng, Y., Wang, G. E. & Zang, Y. Y. Identification and grading of Wuhan commercial center based on POI. *Mod. Urban Res.* 109–116 (2021).

[25] González-Hernández EM, Orozco-Gómez M (2012). A segmentation study of Mexican consumers based on shopping centre attractiveness. Int. J. Retail Distrib. Manag..

[26] Masuyama A (2014). Total locational surplus for facility users distributed continuously along a network. Int. J. Geogr. Inf. Sci..

[27] Mao H (2019). Customer attractiveness evaluation and classification of urban commercial centers by crowd intelligence. Comput. Hum. Behav..

[28] Rajagopal (2011). Determinants of shopping behavior of urban consumers. J. Int. Consumer Market..

[29] Shan ZR, Wu Z, Yuan M (2021). Exploring the influence mechanism of attractiveness on Wuhan's urban commercial centers by modifying the classic retail model. Isprs Int. J. Geo-Inf..

[30] Yan LX, Zang SW, Wang D, Xie DC, Chen Y (2016). Identification and evaluation of Shanghai urban life center system. Urban Plan. Forum.

[31] Yin ZX, Wang D, Yan LX, Zhao BC (2019). Identification and evaluation of living centers in the central urban area of Chongqing. Planners.

[32] Lin Q, Sun F, Wang XM, Liao C, Zhang WX (2019). Research on the ranking system of commercial centers in Beijing based on POI. J. Beijing Normal Univ. Nat. Sci..

[33] Cheng JY, Zhang YB, Wang C (2022). A study on the characteristics of occupancy and residential space in the suburbs of Hefei city based on mobile phone signaling data. J. Huazhong Agric. Univ..

[34] Getis A, Ord JK (1992). The analysis of spatial association by use of distance statistics. Geogr. Anal..

[35] Ord JK, Getis A (1995). Local spatial autocorrelation statistics: Distributional issues and an application. Geogr. Anal..

[36] Sevtsuk A, Kalvo R (2017). Patronage of urban commercial clusters: A network-based extension of the Huff model for balancing location and size. Environ. Plan. B Urban Anal. City Sci..

[37] Ding L, Niu XY, Song XD (2020). Validating gravity model in multi-centre city: A study based on individual mobile trajectory. Acta Geogr. Sin..

[38] Xin L (2022). Recognition of Zhengzhou urban multi-center spatial form based on influence sphere of gravity. Acta Geogr. Sin..

[39] Wei CY (2014). Spatial Behavior and Behavioral Space.

[40] Zhu W, Timmermans H, De W (2006). Temporal variation in consumer spatial behavior in shopping streets. J. Urban Plan. Dev.-ASCE.

[41] Deng Y, Liu S, Wang L, Ma H, Wang J (2010). Field modeling method for identifying urban sphere of influence: A case study on central China. Chin. Geogr. Sci..

[42] Chen YG, Liu JS (2001). Reconstructing Steindl's model: From the law of allometric growth to the rank-size rule of urban systems. Sci. Geogr. Sin..

[43] Chen H, Yang D, Li J, Wu R, Huo J (2020). Distribution characteristics and influencing factors of commercial center and hotspots based on big data: A case of the main urban area of Urumqi City. Prog. Geogr..

[44] Chen PY (2019). Effects of normalization on the entropy-based TOPSIS method. Expert Syst. Appl..

[45] Hu TY, Yang J, Li XC, Gong P (2016). Mapping urban land use by using landsat images and open social data. Remote Sens..

[46] Chen ZQ (2017). A new approach for detecting urban centers and their spatial structure with nighttime light remote sensing. IEEE Trans. Geosci. Remote Sens..

[47] Dolega L, Pavlis M, Singleton A (2016). Estimating attractiveness, hierarchy and catchment area extents for a national set of retail centre agglomerations. J. Retail. Consum. Serv..

[48] Lu FT (2020). Research on identification and spatial differentiation of Hefei city center system based on LBS. J. Anhui Jianzhu Univ..

